# The correlation between vitamin D levels and demographics in patients with gastrointestinal disorders; a cross-sectional study 

**Published:** 2020

**Authors:** Suhaib JS. Ahmad, Ahmed R Ahmed, Jafer Ali, George Macfaul, Matt W Johnson, Aristomenis K. Exadaktylos, Rami Archid, Sami Ahmad, Mohammad Rostami-Nejad, Hamid Mohaghegh-Shalmani, Ravi Madhotra, Kamran Rostami

**Affiliations:** 1 *School of Medicine, University of Buckingham, Buckingham, UK*; 2 *Department of Bariatric and Metabolic Surgery, Imperial College London, London, UK *; 3 *Department of Gastroenterology, Milton Keynes University Hospital, Milton Keynes, UK *; 4 *Department of Gastroenterology, Luton & Dunstable Hospital, Luton, UK*; 5 *Department of Emergency Medicine, Inselspital, University Hospital of Bern, Bern, Switzerland*; 6 *Department of General, Visceral and Transplant Surgery, Eberhard-Karls-University Hospital Tuebingen, Tuebingen, Germany*; 7 *Istishari Private Hospital, Amman, Jordan*; 8 *Gastroenterology and Liver Diseases Research Center, Research Institute for Gastroenterology and Liver Diseases, Shahid Beheshti University of Medical Sciences, Tehran, Iran*; 9 *Department of Gastroenterology Palmerston North Hospital, New Zealand *

**Keywords:** Vitamin D deficiency, 25-hydroxyvitamin D, Sunlight, demographics, IBD, Liver disease, Gastrointestinal disorders

## Abstract

**Aim:**

The aim of the present study was to evaluate vitamin D levels, in correlation with age, body mass index (BMI), gender and ethnicity, in patients with gastrointestinal disorders (GID).

**Background:**

Vitamin D deficiency (VDD) is a global health issue, affecting over 1 billion people. A great body of evidence has shown that it can lead to increased morbidity and mortality. Furthermore, latitude, sedentary lifestyle, limited sunlight exposure, ageing and the presence of comorbidities and chronic illnesses, places patients at an increased risk of VDD.

**Methods:**

305 consecutive patients, with GID, were assessed for vitamin D levels, using a two-step competitive binding immunoenzymatic assay. Patients were then classified as adequate (50-150nmol/l), insufficient (25-50nmol/l) and deficient (<25nmol/l).

**Results:**

62% of the investigated subjects had low vitamin D levels. From this group, 132 patients (43.3%) had insufficient vitamin D levels, 57 (18.7%) had deficient levels and 116 (38%) had adequate levels. Age was not significantly different in the 3 groups (p=0.29). Interestingly, vitamin D levels were significantly lower in men (39.23±23.62) compared to women (50.68±24.46) (p=0.0001). The BMI was significantly higher in patients with insufficient vitamin D levels. Being of Asian ethnicity had a positive influence on vitamin D levels (B=0.076) (p<0.0001). 71.4% of patients, with IBD, and 60% of patients, with abnormal liver function, had low vitamin D levels.

**Conclusion:**

VDD has a high prevalence in patients with GID in particular IBD and liver disease in the United Kingdom. Routine vitamin D testing and supplementations in the case of deficiency and suboptimal level of vitamin D for patients with hepatobiliary, pancreatic, kidney, malabsorptive and restrictive diseases/surgeries is recommended.

## Introduction

 Vitamin D promotes the absorption of calcium, in the proximal intestine, and induces the formation and activation of osteoclast, to function in the mobilization of calcium from bone. It is also well established that vitamin D, together with the parathyroid hormone stimulates the reabsorption of calcium in the distal renal tubules (1). Thus, a shortfall in vitamin D levels can lead to rickets in children, which presents as bowing of the legs, and osteomalacia in adults, which presents as a poorly mineralised skeletal matrix. Low levels of vitamin D can also lead to osteopenia, which can progress to osteoporosis (2). Osteoporosis is defined as a significant reduction in the bone mineral density (T score <2.5) and this reduction significantly increases the risk of fractures (3). It has also been determined that VDD is associated with musculoskeletal pain in adults, cardiovascular diseases, type 2 diabetes mellitus, cancer, autoimmune conditions, depression and an impaired immune system (4, 5).

Sufficient exposure to ultraviolet B radiation is imperative for achieving adequate levels of pre-vitamin D_3_, through the cutaneous endogenous activation of 7-dehydrocholesterol, found in plasma membranes. The pre-vitamin D_3 _then undergoes thermally induced double-bond rearrangement to form vitamin D_3 _(1). An increase in skin pigmentation, ageing and the application of topical sunscreen, curtail the cutaneous production of vitamin D_3_. Other factors that might hinder the number of ultraviolet B photons reaching the earth surface include latitude, season, time of the day and air pollution (6). A model developed by Webb AR et al. revealed that winter sunlight does not promote the synthesis of pre-vitamin D_3 _(7). Subsequently, nutrition and oral supplementations are indispensable for achieving adequate levels of vitamin D. Vitamin D_3 _(cholecalciferol) and Vitamin D_2 _(ergocalciferol) are readily available oral supplements. 

The cutaneous formed or ingested vitamin D is transported to the liver by the vitamin D binding protein (VDBP) to undergo its first hydroxylation. This results in the formation of 25-hydroxyvitamin D3 (25(OH)D_3_). The 25(OH)D_3_, which constitutes the major circulating form of vitamin D, is then transported to the kidneys. In the proximal renal tubule, the 25(OH)D_3_ is then converted into the active form of vitamin D, the 1,25 dihydroxyvitamin D3 (1,25(OH)_2_D_3_). The active 1,25(OH)_2_D_3_ is responsible for the majority of the biological actions of vitamin D (1). The circulating level of 25(OH)D_3 _is used as an indicator of vitamin D status (8). 

Vitamin D is fat-soluble and is also absorbed into the bloodstream, through the gastrointestinal system. Almost three-quarters of the administered vitamin D isabsorbed by the jejunum and ileum, to appear in both the portal and lymphatic systems (9). Disruptions to the normal gastrointestinal function, as a result of infections or diseases, may initially lead to metabolic acidosis, which is well tolerated acutely. However, it can eventually lead to protein catabolism, mineral abnormalities and hormonal alteration, exacerbating any pre-existing deficiencies (10, 11, 12, 13.). In addition to primary pathological processes, the frequently prescribed glucocorticoids are well known to be associated with VDD (14). Furthermore, hypogonadism, decreased physical activity and increased parathyroid hormone production, are recognized risk factors for VDD (15, 16, 17).

The constitutional objective of this cross-sectional study is to expand the understanding of the compelling low vitamin D prevalence amongst patients presenting with disorders affecting the gastrointestinal tract and the accessory digestive organs, and its association with age, sex, BMI and ethnicity. This in turn apprises whether VDD should be routinely screened for and appropriately corrected in all patients with GID. 

## Methods

**Study Population**

455 patients attended the Gastrointestinal Outpatient clinic, in Milton Keynes University Hospital, between September 2015 and July 2016.

Inclusion criteria:

1-Patients presenting with GID.

2-Patients who had their vitamin D level tested as part of the initial investigation at the time of presentation.

305 patients met the inclusion criteria and 150 patients were excluded from the study. 

**Data Collected**

Demographic data including age, height, weight, gender, clinical presentation and diagnosis at admission and the levels of 25-hydroxyvitamin D[25(OH)D], were collected from the study population. Patients were also classified as Asian (Indian, Pakistani, Bangladeshi, Chinese or other Asian), Caucasian, Black (African or Caribbean) and Mix, according to the United Kingdom Census 2011. 

**Measurement of the BMI**

The body mass index (BMI) was derived from the weight and height of the individual. Age and sex were taken into account and the results were expressed in units of kg/m^2^. A BMI of <18.5 was considered as underweight, 18.5-24.9 ideal, 25-29.9 Overweight, 30-34.9 obese, 35-39.9 severely obese, 40-49.9 morbidly obese, >49.9 super obese. These BMI values are according to the World Health Organisation. 

**Measurement of vitamin D**

Vitamin D levels were measured using a two-step competitive binding immunoenzymatic assay (Diasorin LIAISON 25 OH Vitamin D Total Assay (K112725) Manufactured by Diasorin, Inc). 

Vitamin D levels were classified into 3 major groups according to the Milton Keynes University Hospital reference ranges. Adequate (50-150 nmol/l); Insufficient (25-50nmol/l) and Deficient (<25nmol/l).

**Statistical analysis**

**Table 1:** Prevalence of symptoms in patients with different levels of vitamin D

Symptoms	Total 305 patients; N(%)	Proportion of patients with insufficient and deficienct vitamin D levels who exhibit the symptom; N(%)	Proportion of patients with adequate vitamin D levels who exhibit the symptom; N(%)
Abdominal pain	50(16.4)	29(15.4)	21(18.1)
Diarrhoea	29(9.5)	14(7.4)	15(12.9)
PR bleeding	10(3.3)	9(4.8)	1(0.9)
Dyspepsia	27(8.9)	9(4.8)	18(15.5)
Weight loss	11(3.6)	8(4.2)	3(2.6)
Nausea/Vomiting	13(4.3)	10(5.3)	3(2.6)
Constipation	11(3.6)	5(2.6)	6(5.2)
Reflux	4(1.3)	3(1.6)	1(0.9)
Bloating	4(1.3)	3(1.6)	1(0.9)
Anal pain	1(0.3)	1(0.5)	0(0.0)
Dysphagia	2(0.7)	0(0.0)	2(1.7)

**Table 2:** Mean BMI and Age for patients with different levels of vitamin D

Vitmain D	Reference Range	Number in study	Age (Mean±Std. Deviation)	BMI (Mean±Std. Deviation)
Adequate	50-150nmol/L	116 (22 male/94 female)	54.23 ±17.65 years	25.60 ±5.82 Kg/m^2^
Insufficient	25-50nmol/L	132 (54 male/78 female)	51.45 ±17.28 years	27.52 ±5.23 Kg/m^2^
Deficient	<25nmol/L	57 (27 male/30 female)	50.40 ±16.13 years	26.21 ±5.67 Kg/m^2^
Total	-	305	52.31 ±17.23 years	26.55 ±5.59 Kg/m^2^

IBM SPSS version 22.0. Armonk, NY was utilised to perform descriptive and bivariate statistical analysis. Departures from normality were detected and normally distributed data were presented as mean ±std. deviation. The one-way analysis of variance (ANOVA) was used to test the difference between the means of three or more independent groups. Post-hoc testing (Tukey´s HSD) was performed to test for any differences between each of the variables. Pearson Correlation was used to measure the strength and direction of linear relationships of two continuous variables. A Chi-Square test was used to evaluate if the categorical variables, within the population, are associated. The parametric t-test was used to compare the means of two independent groups on the same continuous, dependent variable. Linear regression was the statistical test of choice for modeling the relationship between dependent and independent variables. A p<0.05 was regarded as statistically significant. 

## Results

**The prevalence of low vitamin D levels stood at 62%**

*Clinical association*

Symptoms amongst patients presenting to the outpatient clinic with GID, are demonstrated in table 1. 

*Diagnosis of patients presenting to the outpatient clinic*

Patients were diagnosed with a wide range of conditions, affecting the gastrointestinal tract and the accessory organs of digestion. 94(30.8%) patients had deranged liver function tests (LFTs), of whom 60.0% had low vitamin D levels. In addition, 35(11.5%) patients had IBD (crohn’s disease, ulcerative colitis, collagenous colitis, lymphocytic colitis) of whom 71.4% had either vitamin D deficiency or insufficiency. Furthermore, 65.2% of the 23(7.5%) patients presenting with gluten intolerance, had low vitamin D levels. Patients also presented less commonly with anaemia 21(6.6%), coeliac disease 18(5.9%), lactose intolerance 10(3.3%), barrett’s oesophagus 10(3.3%), reflux disease 10(3.3%), diverticular disease 9(3%), pancreatic lesion/pancreatitis 7(2.3%), oesophagitis 4(1.3%), IBS 4(1.3%), gastritis 3(1.0%), MS 2(0.7%), gastric ulcer 2(0.7%), gastrointestinal stromal tumours (GIST) 2(0.7%), gilbert 2(0.7%), cytomegalovirus 1(0.3%), SLE 1(0.3%), sarcoidosis 1(0.3%), hyperthyroidism 1(0.3%), renal transplant 1(0.3%), hemorrhoids 1(0.3%), H pylori infection 1(0.3%), duodenal ulcer 1(0.3%) and rectal ulcer 1(0.3%). All of the presenting diseases were more common in patients with vitamin D deficiency/insufficiency except for barrett’s disease, reflux disease, diverticular disease, irritable bowel syndrome (IBS), gastritis, multiple sclerosis (MS) and hemorrhoids.

*Vitamin D levels in correlation with BMI*

**Figure 1:**
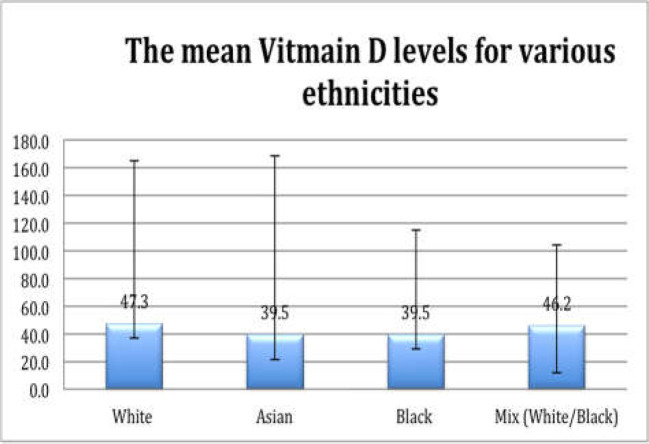
Figure illustrating the relationship between vitamin D levels and BMI

A third of patients had an Ideal BMI (33.9%), 30% were overweight and 21.8% were obese. 6.5%, 7.2% and 0.7% of patients were underweight, severely obese and morbidly obese respectively. Of the patients with adequate vitamin D levels, 29.9% had ideal weight, 23.9% were overweight, 20.9% were obese, 12.7% underweight, 11.2% severely obese and 1.5% were morbidly obese. Of the patients with insufficient vitamin D levels, 32.8% had ideal weight, 31.3% were overweight, 13.4% were severely obese, 19.4% obese and 3% were underweight. Of the patients with deficient vitamin D levels, 35.1% had ideal weight, 33.3% were overweight, 22.8% were obese, 5.3% overweight and 3.5% were underweight. The BMI was not significantly different between men (26.25±5.54) and women (26.70±5.63; P=0.5). There was however, a significant difference seen between the BMI of the 3 vitamin D groups; insufficient (n=132), deficient (n=57), adequate (n=116) vitamin D (p<0.023). Post hoc analysis showed a significant difference between the BMI of the insufficient and adequate vitamin D groups, where the BMI was significantly higher in the insufficient group (Table 2). Pearson correlation coefficient demonstrated a significant weak negative correlation between vitamin D level and BMI (r=-0.13, p=0.02). According to the coefficient of determination (r^2^= 0.017), BMI can predict vitamin D level only in 1.7 percent of the cases (Figure 1). 

*Vitamin D levels in correlation with ethnicity*

**Figure 2:**
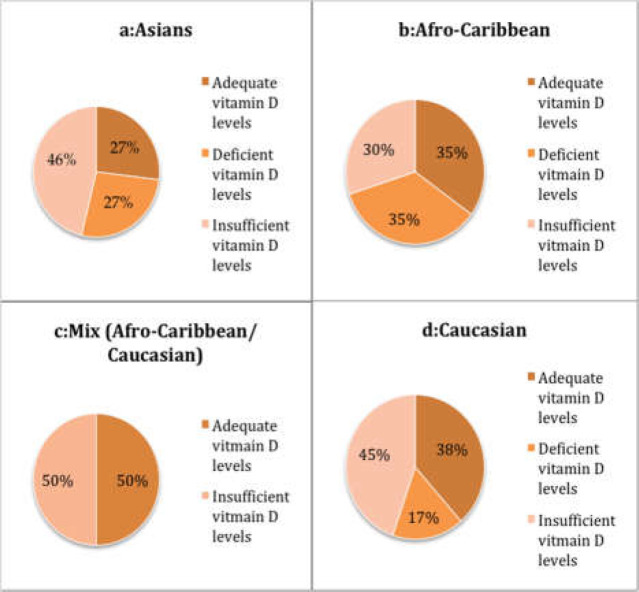
Vitmain D levels among various ethnicities; (a) Asians (b) Afro-Caribbean (C) Mix (Afro-Caribbean/ Caucasian) (d) Caucasian

Of the 305 patients, 275 were of Caucasian origin, 2 of mix (Caucasian / Afro-Caribbean) origins, 20 Afro-Caribbean and 26 were of Asian origins. 12(46.2%) out of the 26 Asians had insufficient vitamin D levels and 7(26.9%) had deficient levels (figure 2a). 7 out of the 20 Afro-Caribbean patients had deficient vitamin D levels and 6 had insufficient vitamin D levels (Figure 2b). 50% of the 2 Mix race patients had insufficient vitamin D levels (Figure 2c). Out of the 257 Caucasian patients, 115(44.7%) had insufficient vitamin D levels and 43(16.7%) had deficient vitamin D levels (Figure 2d). 

**Figure 3:**
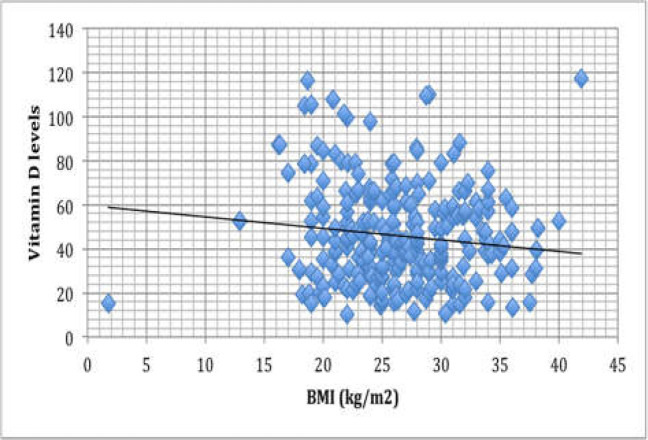
The mean vitamin D levels for various ethnicities

The mean vitamin D level according to ethnic origin was mentioned in figure 3. As data showed, the mean vitamin D level for the Caucasian population (47.3±70.1) was higher than other groups. The linear regression model demonstrated a slight positive influence of the Asian ethnicity on vitamin D levels (Unstandardized Coefficient B value=0.076; P=0.000.

## Discussion

Over 1 billion people worldwide are deemed to have VDD (17). People living in Europe appear to be at a substantially greater risk due to the scarcity of vitamin D-fortified food. The sunniest areas also experienced VDD, where most of the skin is shielded from the sun, such as in Saudi Arabia and the United Arab Emirates (18-20). The result of this study revealed that pre-existing GID, sex, BMI and ethnicity can impact vitamin D levels**. **The prevalence of VDD in the current study (62%) was significantly higher than those reported by other studies, conducted on the general healthy population (21, 22). Certain presenting symptoms such as abdominal pain, nausea/vomiting and PR bleeding were more common amongst patients with insufficient and deficient vitamin D levels. However, these upper and lower GI complaints can be a result of a wide range of physiological and functional disorders. Thus, it would be puzzling to link these symptoms directly to VDD. However, the symptoms can be used to make a diagnosis, which can in turn provide us with trusted information on whether the patient is at risk of vitamin D deficiency. 

Vitamin D absorption is determined by 6 different factors (23, 24, 25. ). Firstly, the intake of vitamin D should be adequate, especially in individuals with an impaired skin ability to synthesize sufficient quantities of vitamin D. This was demonstrated in this study, where most patients, with suboptimal vitamin D status, presented with GID that can reduce the appetite such as gastritis, acute pancreatitis and gastroesophageal reflux disease.

In the case of coeliac disease, the high rates of vitamin D deficiency (55.5%) might be due to the loss of appetite, residual absorption issue or due to the inadequate intake of vitamin D, due to the strict gluten free diet products. Secondly, an unblemished intestine, pancreas and liver are indispensable for the delivery of the lipase and bile acids, required for vitamin D absorption. Gastrectomies, gastric bypass, pancreatic insufficiency, small intestine disorders and an anomalous biliary tract, are all regarded as risk factors for having low vitamin D levels. Thirdly, the impaired body’s ability to metabolize vitamin D into active compounds has also been associated with VDD. Fourthly, it is imperative that the enterohepatic pathway is fully functional to prevent any losses. Vitamin D is first conjugated and secreted in bile prior to its reabsorption by the small intestine. Fifthly, liver diseases debilitate the body’s ability to transport vitamin D metabolites to tissues. Lastly, a diseased or surgically altered intestine might not respond ordinarily to vitamin D metabolites absorption in relation to calcium and phosphate levels. A shortened post-surgical bowel (especially with ileo-caecal valve resection) can increase the small bowel transit time, thereby reducing the time available for vitamin D absorption. The aforementioned risk factors were confirmed in this study, where out of 100% of patients presenting with pancreatic lesions/pancreatitis, 71.4% of patients with IBD and 60.0% with abnormal liver function tests had suboptimal vitamin D levels. 

Males (39.23±23.62) had significantly lower vitamin D levels compared to females (50.68±24.46) (p<0.0001), contradicting results noted elsewhere (26-28). This might be elucidated by the fact that females receive significantly more sunlight exposure, compared to males, due to less skin covering. However, this is not the case in countries where women cover their whole body as part of a religion or a tradition such as in Iran and the United Arab Emirates. Furthermore, the lower vitamin D status in men might be due to the fact that men exceed women in alcohol consumption and high-volume drinking (29). Thus, men are more likely to be malnourished and have more severe functional impairment and tissue damage compared to females. 

This study demonstrated a significant negative weak correlation between vitamin D levels and BMI (r=-0.13, p=0.02). This is in accordance with many other studies (30). Vitamin D is a fat-soluble hormone. Subsequently, individuals with a higher percentage of adipose tissue might be the target for vitamin D sequestration, leading to decreased circulating levels of the hormone. Other studies have also noted an inversely proportional relationship between high lipid profiles (associated with an increased BMI) and the circulating vitamin D levels (31). A study published by Brock et al., suggested that a BMI of > 30 kg/m2 should be regarded as a major risk factor for having low vitamin D levels (32). This study demonstrated that BMI could only predict low vitamin D levels in 1.7 percent of the cases. As a result, BMI should be regarded as a potential risk factor, but should not be utilized as a predictor of vitamin D levels. 

In patients presenting with disorders affecting the gastrointestinal system, being Asian has a slight positive influence on vitamin D levels. This goes against the findings of several older studies, conducted on the general healthy population, which regarded Asian ethnicity as a risk factor for having low vitamin D levels (33, 34). It is possible that this may reflect the successful outcome of several high profile public health campaigns over the last decade that have come to address the common prevalence of vitamin D deficiency among Asian families. This has led the Committee on Medical Aspects of Food Policy (COMA) in the United Kingdom to advise Asian children to take vitamin D supplements throughout the first five years of life (33). As a result, awareness about the deficiency among Asians has increased. The Asian population, described in this study, presented mostly with chronic GID. Such an increase in health consciousness might have prompted the Asian patients to use dietary supplements, sooner and more liberally, to maintain health or prevent progression of disease. Furthermore, Most of the Asian patients experienced pain as part of their presenting symptoms and the pain symptom has been shown to be associated with physician-advised vitamin D supplementation (35). Thus, most patients presenting to the outpatient clinics will have already seen their GPs, where they might have already been advised to take supplementations. **Screening for VDD and supplementations in patients with GID.**

*Screening*

Targeted screening of vitamin D levels in patients presenting with GID, enables patients, who need to be treated, to be identified and treated with an accurate and effective dose. Since kidneys are greatly responsible for the conversion of vitamin D into its active metabolite 1,25(OH)_2_D, all patients, presenting with chronic kidney disease, should also be screened for vitamin D deficiency (5). In addition, patients with generalized symptoms such as pains, myalgias and weaknesses, should also be screened for vitamin D deficiency since these symptoms are commonly misdiagnosed with other medical conditions such as chronic fatigue and fibromyalgia (36).

Due to the high VDD prevalence in patients with GID, it has been recommended that all patients presenting with the GID inflammatory bowel disease, irritable bowel syndrome, liver disease (including hepatitis, cirrhosis and liver lesions), pancreatic diseases (including acute and chronic pancreatitis and pancreatic lesions), coeliac disease, diverticular disease, gastroesophageal reflux disease and diseases/surgeries leading to malabsorption/restriction (eg: Gastric bypass/gastric sleeve/cystic fibrosis), should be screened for vitamin D levels. 

*Supplementation*

Supplementations are regarded to be safe and cheap (37). Many studies suggest that a vitamin D concentration of > 30 ng/mL is required to maintain adequate suppression of the parathyroid hormone production (38). Achieving a concentration of >30 ng/mL endures many challenges. Firstly, most physicians are reluctant to prescribe large doses of vitamin D. Secondly,adequate intake does not always lead to normal levels, especially in predisposed individuals or those at risk (39). Both D_2 _(ergocalciferol) and D_3_ (cholecalciferol) are widely accepted as vitamin D supplements. Both types are regarded to be equally effective at dosages of 50,000 IU. However, it is suggested that D_3_ has a longer half-life. As a result, less frequent dosages of D_3 _are required, leading to an enhanced patient adherence and compliance (40, 41). 

-Vitamin D deficiency (levels less than 25nm/l): treatment include a loading of 50,000IU weekly for 6 weeks, followed by a lifelong maintenance dose of 50,000IU a month. 

-Vitamin D insufficiency (25-50nmol/l): maintenance dose of 50,000IU a month should be started immediately, without the use of loading doses (41). 

-Sufficient vitamin D levels (greater than 50nm/l): advice on measures to prevent vitamin D deficiency including sufficient and safe UV exposure (exposure of arms and legs for 5-30 minutes, between 10am and 3pm, twice a week) and a balanced diet (42). The advice also entails oral supplementations, when there is a paucity of UV exposure or when there is a scarcity in nutritional resources.

*Monitoring*

*Vitamin D levels should be checked after 3-6 months. Afterwards, routine monitoring of serum vitamin D levels, during the long-term supplementation, is not required.

-If levels are greater than 50nm/l and there are no signs of hypercalcaemia, the maintenance dose should be continued. 

-If levels are below 50nmol/l, check for possible causes including compliance, drug interaction and if no cause is found, consider higher doses of vitamin D.

*Adjusted serum calcium levels should be checked 1 month after commencing vitamin D supplementation, in cases where primary hyperparathyroidism has been unmasked. 

-If calcium levels are high, assess hydration state and admit to hospital if necessary and if the patient was taking calcium supplements, advise them to stop taking them. 

-If calcium levels are normal, do not recommend long-term calcium supplements and if the patient was taking calcium supplements, advise them to stop taking them.

-If calcium levels are low, treat according to severity and admit to hospital if necessary. For chronic hypocalcaemia, advise adequate calcium intake and if this is not possible, advise the use of over-the-counter calcium supplements, containing 1-2g of calcium, in patients with inadequate calcium intake, and this might be required long term. If the patient was already taking supplements, a specialist referral should be made (41).

Preparations containing vitamin D and calcium (such as Calcichew-D_3_®), should not be used to treat patients with vitamin D deficiency, since they contain low doses of vitamin D and might result in hypercalcaemia in patients requiring high doses of vitamin D. 

**Is targeted screening and supplementation cost-effective in patients, presenting with GID?**

This study has demonstrated that patients, with certain GID, are predisposed to low vitamin D levels. When discussing the cost effectiveness of targeted screening and supplementations, in patients with GID, we need to learn a lesson from other fields. For instance, when considering type 2 diabetes mellitus, those at risk are screened and the general population is given advice on healthy lifestyle. In vitamin D, aggressive screening and supplementation strategies need to be implemented for those at risk. The US Veterans Medical Centre conducted a cost-analysis, comparing the inpatient and outpatient costs for individuals, who had received at least one vitamin D test and subsequent screenings. Results showed a negative correlation between vitamin D levels and medical costs. In addition, their study also demonstrated lower costs for patients, with two or more follow up screenings, indicating that subsequent monitoring can also lower medical costs (43). Furthermore, a univariate analysis, published by Matthew et al. in 2012, revealed that low vitamin D levels are associated with increased hospital stay, surgical intensive care unit costs and mortality (44). Moreover, cost-benefit analysis has demonstrated that both universal supplementation and screening for vitamin D levels, amongst patients at risk are cost effective and this study has revealed certain GIDs to be a risk factor for having low vitamin D levels (45). 

**Limitations of the study**

The main limitation associated with most vitamin D studies is the lack of accord about the definition of VDD,and where the cut off points are. In addition, studies cited in this study had different methodologies and were performed on divergent study populations. Some of the studies cited may not have collected their data in accordance with the standard guidelines, giving rise to inaccurate data. This highlights the importance of standardizing methodologies, in order to ensure accurate results. One further limitation of this study was the unknown historical vitamin D supplementation status of patients, at the time of presentation.

To conclude, VDD is prevalent among patients with GID. These results are achieved from looking at the negative impact GID has on vitamin D absorption and metabolism. High BMI was shown to be a risk factor, but not a useful predictor in this cohort. Also, being male did increase the risk of VDD. All patients presenting with GID that can affect vitamin D absorption, transport or metabolism, should be evaluated and tested for VDD. Once detected, Vitamin D supplementations should be given in an acceptable form dependent on the underlying GI disorder, to achieve normal levels of 25(OH)D calcium and PTH levels.

## Conflict of interests

The authors declare that they have no conflict of interest.
